# Diversity priors for learning early visual features

**DOI:** 10.3389/fncom.2015.00104

**Published:** 2015-08-12

**Authors:** Hanchen Xiong, Antonio J. Rodríguez-Sánchez, Sandor Szedmak, Justus Piater

**Affiliations:** Intelligent and Interactive Systems Group, Institute of Computer Science, University of InnsbruckInnsbruck, Austria

**Keywords:** restricted Boltzmann machine, diversity prior, V1 simple cell, inhibition, Markov networks

## Abstract

This paper investigates how utilizing diversity priors can discover early visual features that resemble their biological counterparts. The study is mainly motivated by the sparsity and selectivity of activations of visual neurons in area V1. Most previous work on computational modeling emphasizes selectivity or sparsity independently. However, we argue that selectivity and sparsity are just two epiphenomena of the diversity of receptive fields, which has been rarely exploited in learning. In this paper, to verify our hypothesis, restricted Boltzmann machines (RBMs) are employed to learn early visual features by modeling the statistics of natural images. Considering RBMs as neural networks, the receptive fields of neurons are formed by the inter-weights between hidden and visible nodes. Due to the conditional independence in RBMs, there is no mechanism to coordinate the activations of individual neurons or the whole population. A diversity prior is introduced in this paper for training RBMs. We find that the diversity prior indeed can assure simultaneously sparsity and selectivity of neuron activations. The learned receptive fields yield a high degree of biological similarity in comparison to physiological data. Also, corresponding visual features display a good generative capability in image reconstruction.

## 1. Introduction

**Figure 1:**
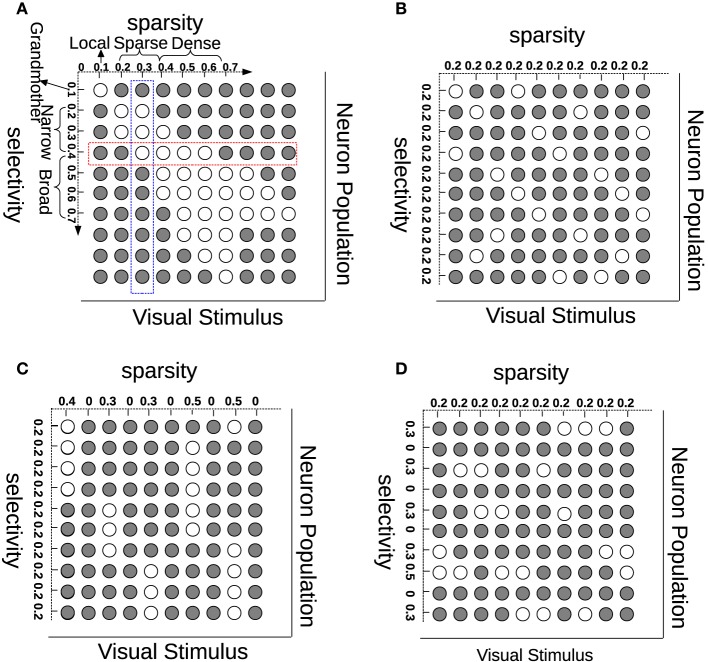
**Understanding sparsity and selectivity**. White circles indicate activations while gray circles denote inactivations. **(A)** Explaining the concepts of sparsity and selectivity; **(B)** An example of good sparsity and good selectivity; **(C)** An example of good selectivity but bad sparsity; **(D)** An example of good sparsity but bad selectivity. See text for further description.

Much has been advanced in the knowledge of the brain in the last century since the foundation of modern neuroanatomy by Ramón y Cajal (Ramón y Cajal, 1888, 1904; Jones, 2007). The work of HUBEL and WIESEL (1959) was the first breakthrough in the understanding of simple cells in area V1 of the visual cortex. V1 simple cells perform an early stage processing of the visual input from the retina and the lateral geniculate nucleus (LGN). One important property of V1 simple cells is that their receptive fields are *selective* in terms of location, orientation, and frequency, which can be modeled by Gabor filters. Another characteristic on V1 simple cells is that their activation pattern—when analyzed as a population—is sparse (Field, 1994). Selectivity (also referred to as “lifetime sparseness” by Willmore and Tolhurst, 2001) is related to a neuron having a response only to a small number of different (although similar) stimuli and providing a much lower response to other (usually very different) stimuli. Sparsity (or “population sparseness” by Willmore and Tolhurst, 2001) is a term expressing that the fraction of neurons from a population that is activated by a certain stimulus should be relatively small. Selectivity and sparsity would be due to a redundancy-reduction mechanism, where the visual cortex has evolved to encode visual information as efficiently as possible (Barlow, 1989). This *sparse coding* would then enhance coding efficiency, and when tested, leads in fact to Gabor-like representations (Olshausen and Field, 1996). Although sparse coding has been very successful at generating receptive fields similar to those of simple cells, sparsity does not necessarily imply selectivity (Willmore and Tolhurst, 2001). In addition to this, recent multi-unit neurophysiological recordings found that just maximizing sparsity does not correlate with visual experience, suggesting that coding efficiency is also due to lateral, recurrent and feedback connections for the purpose of resolving ambiguities (Berkes et al., 2009). In order to show the (lack of) relationship between sparsity and selectivity, we illustrate these concepts in Figure 1A. Each row (red) in this figure represents how one neuron selectively responds to different visual stimuli while each column (blue) describes how many neurons are activated by one stimulus. Although selectivity and sparsity can be related at their average values, they are not necessarily correlated: Selective neurons do not ensure sparse neuron coding (Figure 1C); similarly, sparsely activated neurons are not necessarily narrowly selective (Figure 1D).

**Figure 2:**
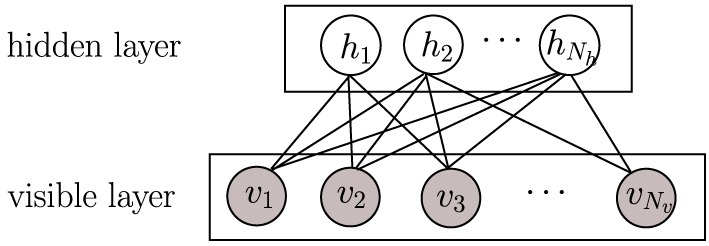
**A graphical model of a restricted Boltzmann machine (RBM)**. Gray circles represent observed variables while empty circles are hidden variables.

Another hypothesis on how to achieve coding efficiency is dependence minimization, which can be achieved applying *independent component analysis* (ICA) (Hyvärinen and Oja, 2000). ICA is a dimensionality reduction methodology widely used in signal processing for decomposing a compound signal into their components (or so-called bases) that are as independent as possible. In ICA, independence maximization is achieved by pursuing extrema of the kurtosis (a measure of function “peakedness”) of each components' distribution. Applying ICA on natural images has also produced receptive fields like those of V1 simple cells (Bell and Sejnowski, 1997; van Hateren and van der Schaaf, 1998). Be either ICA or sparse coding, in the end, they are two successful learning strategies that can learn primary visual cortex-like receptive fields (Olshausen and Field, 1996; van Hateren and van der Schaaf, 1998). Another successful learning strategy at emulating the hierarchical architecture of the brain is *deep learning* (Bengio, 2009; LeCun et al., 2015), which is usually constructed with a stack of restricted Boltzmann machines (RBMs). RBMs have recently attracted increasing attention due to its successes in learning representations (Hinton, 2002; Hinton and Salakhutdinov, 2006). In RBMs, there is no connection among hidden units (Figure 2), which makes inference and learning of RBMs quite easy and fast. That means that given some visible data, all hidden units are conditionally independent from each other (see Section 2.2). Even so, RBMs provide a nonlinear coding of natural images, which goes beyond sparse coding or ICA. However, the capability of RBMs is still limited when learning receptive fields similar to those of V1 simple cells. When RBMs are trained on natural images, many learned features can be rather distributed, unlocalized and repeated, which is far from the (selective and sparse) nature of the learning task. Prior work has exploited different strategies to adapt RBMs toward learning selective or sparsely-activated neurons (Lee et al., 2007; Goh et al., 2010; Luo et al., 2011) on visual inputs. Meanwhile, most of those works focus on either one property, thus not ensuring sparsity and selectivity simultaneously in the resulting emulated neurons, which as mentioned before may be suboptimal for coding efficiency.

Empirically, neither sparse coding nor ICA can yield both, good selectivity and sparsity simultaneously (Willmore and Tolhurst, 2001). In this paper, we propose a novel hypothesis to interpret the selectivity and sparsity of neuron activations through the *diversity of neurons' receptive fields*. Based on the analysis exposed above, we can see that the effect of sparsity is to better differentiate neurons, while the goal of selectivity is to avoid “over-tolerant” neurons, thus both aimed at reducing ambiguities. We propose that, in order to reach bot—high degrees of neural population sparsity and individual neuronal selectivity—we need one condition: diverse receptive fields. To the best of our knowledge, the diversity of receptive fields (features) has rarely been exploited to guide learning, even though it has been achieved unintentionally in several existing models. By contrast to conventional models, we use diversity as a starting point instead of as a result. An earlier pioneering work focusing on the importance of diversity in neural coding was presented by Padmanabhan and Urban (2010).

We argue that selectivity and sparsity of neurons' activations can be seen as two epiphenomena of the diversity of receptive fields. To verify this hypothesis, we impose a *diversity prior* on the inter-weights within the RBMs when learning simple neurons' receptive fields from natural images. This prior will introduce a bias over the inter-weights toward higher degrees of *sum similarity minimization*. The prior indirectly coordinates neurons' activations by diversifying the inter-weights within the RBMs, which would mimic the effect of inhibition. It is worth noting that the prior is only employed in the learning phase, yet its implicit effect on coordinating neurons' activations will remain after learning. In this sense, the diversity prior is in line with the influence of inhibitory interneurons (King et al., 2013) (see Section 2.3 for more details). It should be finally noted that we do not consider an RBM (even if trained with diversity priors) as a full biologically-plausible model of V1 simple cells, since we are not considering many other aspects and properties of simple cells, e.g., contrast normalization, contrast adaption, etc. The purpose of our study is to verify and advocate for using *diversity* as a new principle in order to guide the learning of more similar primary visual cortex cell receptive fields.

## 2. Materials and methods

In this section, we describe our basic experimental setup, which includes the construction of visual stimulus data, the restricted Boltzmann machine (RBM), and the proposed prior for training. For the RBM, a brief introduction of the model and its probabilistic properties is provided in Section 2.2. Readers are referred to Hinton (2002) for a more detailed and deeper study.

### 2.1. Images

The benchmark database from Olshausen and Field (1996)^1^ was used in this paper. This database consists of 10 natural images, which were preprocessed with a pseudo-whitening filter, which flattens the spectrum of natural images by rescaling Fourier coefficients. This step is commonly applied (Olshausen and Field, 1996; Willmore and Tolhurst, 2001), and to some extent is similar to retinal processing. Alternatively, a similar preprocessing function is the log transform, which is more often used in ICA (van Hateren and van der Schaaf, 1998). Then, 100,000 small patches (size 14 × 14) were extracted from random positions of the 10 whitened images. Furthermore, a sigmoid function was applied to the pixel intensities to fit their values into the range [0, 1]. In addition, the patches with variances smaller than 0.1 were filtered out in order to accelerate training.

### 2.2. Restricted Boltzmann machines

The restricted Boltzmann machine (RBM) is a two-layer, bipartite Markov network, which is a “restricted version” of the Boltzmann machine with only inter-connections between a hidden layer and a visible layer. RBMs have been recently rather popular in constructing deep neural networks (DNNs) (Hinton and Salakhutdinov, 2006). A graphical model of an RBM is presented in Figure 2. Input data is binary and *N*_*v*_ dimensional; they are fed into *N*_*v*_ units in the visible layer **v**. The *N*_*h*_ units in the hidden layer **h** are stochastic binary variables, i.e., $\text{v}\in {\left\{0,1\right\}}^{N_{v}}$, $\text{h}\in {\left\{0,1\right\}}^{N_{h}}$. The joint probability of {**v, h**} is:

(1) $$\begin{array}{l} p\left(\text{v},\text{h}\right)=\frac{1}{\text{Z}}\exp\left(-E\left(\text{v},\text{h}\right)\right)\;E\left(\text{v},\text{h}\right)=-{\text{v}}^{⊤}\text{Wh}-{\text{h}}^{⊤}\text{b}-{\text{v}}^{⊤}\text{c} \end{array}$$

where $\text{W}\in {\mathbb{R}}^{N_{v}\times N_{h}}$ is the matrix of symmetric weights, $\text{b}\in {\mathbb{R}}^{N_{h}\times 1}$ and $\text{c}\in {\mathbb{R}}^{N_{v}\times 1}$ are biases for hidden units and visible units, respectively. $Z\;=\;\sum_{v,h}\exp(-E(v,h))$ is the partition function for normalization. In our experiment, to fit the size of small image patches, *N*_*v*_ is equivalent to 196, and *N*_*h*_ is 200, i.e., 200 hidden units. Because of the restricted connections in RBMs, hidden units *h*_*j*_ are conditionally independent of each other given the visible data **v**,

(2) $$\begin{array}{l} p\left(\text{h}|\text{v}\right)=\underset{j}{\prod }p\left(h_{j}|\text{v}\right)\;p\left(h_{j}=1|\text{v}\right)=𝒮\left({\text{v}}^{⊤}{\text{W}}_{\cdot j}+b_{j}\right) \end{array}$$

and similarly, visible units *v*_*i*_ are conditionally independent of each other given **h**.

(3) $$\begin{array}{l} p\left(\text{v}|\text{h}\right)=\underset{i}{\prod }p\left(v_{i}|\text{h}\right)\;p\left(v_{i}=1|\text{h}\right)=𝒮\left({\text{W}}_{i\cdot }\text{h}+c_{i}\right) \end{array}$$

where **W**_*i*·_ and **W**_·*j*_ denote the *i*th row and *j*th column of matrix **W**, *b*_*j*_ and *c*_*i*_ are the *j*th and *i*th entry of vector **b** and **c**, respectively. 𝒮(·) is the logistic function $𝒮\left(x\right)=\frac{1}{1+\exp\left(-x\right)}$. Given training data $𝒟={\left\{{\text{v}}^{\left(l\right)}\right\}}_{l=1}^{L}$, an RBM can be learned by maximizing the average log-likelihood of 𝒟:

(4) $$\begin{array}{l} {\text{W}}^{*}=\arg\underset{\text{W}}{\max}𝓛(𝒟)=\arg\underset{\text{W}}{\max}\frac{1}{L}\overset{L}{\underset{l=1}{\sum }}\left(\log\underset{\text{h}}{\sum }p\left({\text{v}}^{\left(l\right)},\text{h}\right)\right) \end{array}$$

Since the log-likelihood is concave with respect to **W, b, c** (Koller and Friedman, 2009, Chapter 20), based on Equation (1), *gradient ascent* can be applied on Equation (4) by computing the gradient of 𝓛(𝒟) with respect to **W, b, c** as:

(5) $$\begin{array}{rcl} \nabla_{\text{W}}𝓛(𝒟) & = & \frac{1}{L}\overset{L}{\underset{l=1}{\sum }}\left[{𝔼}_{{\text{v}}^{\left(l\right)}\in 𝒟,\text{h}\sim p\left(\text{h}|{\text{v}}^{\left(l\right)}\right)}\left({\text{v}}^{\left(l\right)}{\text{h}}^{⊤}\right)-{𝔼}_{\text{v},\text{h}\sim p\left(\text{v},\text{h}\right)}\left(\text{v}{\text{h}}^{⊤}\right)\right] \end{array}$$

(6) $$\begin{array}{rcl} \nabla_{\text{b}}𝓛(𝒟) & = & \frac{1}{L}\overset{L}{\underset{l=1}{\sum }}\left[{𝔼}_{\text{h}\sim p\left(\text{h}|{\text{v}}^{\left(l\right)}\right)}\left(\text{h}\right)-{𝔼}_{\text{h}\sim p\left(\text{v},\text{h}\right)}\left(\text{h}\right)\right] \end{array}$$

(7) $$\begin{array}{rcl} \nabla_{\text{c}}𝓛\left(\rfloor \right) & = & \frac{1}{L}\overset{L}{\underset{l=1}{\sum }}\left[{𝔼}_{{\text{v}}^{\left(l\right)}\in 𝒟}\left({\text{v}}^{\left(l\right)}\right)-{𝔼}_{\text{v}\sim p\left(\text{v},\text{h}\right)}\left(\text{v}\right)\right] \end{array}$$

where 𝔼_*p*_(·) denotes the expected values with respect to *p*. Obviously, the first terms in Equations (5–7) are easy to compute with **v**^(*l*)^ from 𝒟 and **h** inferred using Equation (2). However, the sampling **v, h** ~ *p*(**v, h**) in the second term of Equation (5) makes learning practically infeasible because it requires a large number of Markov chain Monte Carlo (MCMC) iterations to reach equilibrium. Fortunately, we can compute an efficient approximation to the exact gradient: contrastive divergence (CD), which works well in practice (Hinton and Salakhutdinov, 2006). By using CD_*k*_, only a small number of *k* steps are run in block Gibbs sampling (usually *k* = 1), and Equation (5) can finally be approximated as

(8) $$\begin{array}{l} \nabla_{W}\hat{𝓛}\left(𝒟\right)=\frac{1}{L}\sum_{l=1}^{L}[v^{\left(l\right)}p{\left(h^{(l)+}|v^{\left(l\right)}\right)}^{⊤}-p(v^{(l)-}|h^{(l)+})\\ p{\left(h^{(l)-}|v^{(l)-}\right)}^{⊤}] \end{array}$$

(9) $$\begin{array}{rcl} \nabla_{\text{b}}\hat{𝓛}\left(𝒟\right) & = & \frac{1}{L}\overset{L}{\underset{l=1}{\sum }}\left[p\left({\text{h}}^{\left(l\right)+}|{\text{v}}^{\left(l\right)}\right)-p\left({\text{h}}^{\left(l\right)-}|{\text{v}}^{\left(l\right)-}\right)\right] \end{array}$$

(10) $$\begin{array}{rcl} \nabla_{\text{c}}\hat{𝓛}\left(𝒟\right) & = & \frac{1}{L}\overset{L}{\underset{l=1}{\sum }}\left[{\text{v}}^{\left(l\right)}-p\left({\text{v}}^{\left(l\right)-}|{\text{h}}^{\left(l\right)+}\right)\right] \end{array}$$

where **h**^(*l*)+^ denotes the inferred hidden vector from the *l*th observed data point **v**^(*l*)^ (using Equation 2), and **v**^(*l*)−^, **h**^(*l*)−^ are vectors after one-step block Gibbs sampling (using Equations 2, 3 and again Equation 2).

### 2.3. Imposing a diversity prior

In RBMs, columns of **W** are basis images, with which **v** can be reconstructed from **h**. To some extent, they can also represent neurons' receptive fields. To this end, a natural choice of biasing parameters is to diversify the columns of **W** as much as possible. The way in which we approach diversification is minimizing *square cosine similarities* among columns of **W**:

(11) $$\arg\underset{\text{W}}{\max}\sum_{j=1}^{N_{h}}\sum_{k\neq j}^{N_{h}}{\left\|\frac{W_{\cdot ,j}^{⊤}W_{\cdot ,k}}{\left||W_{\cdot ,j}||||W_{\cdot ,k}|\right|}\right\|}^{2}$$

**Figure 3:**
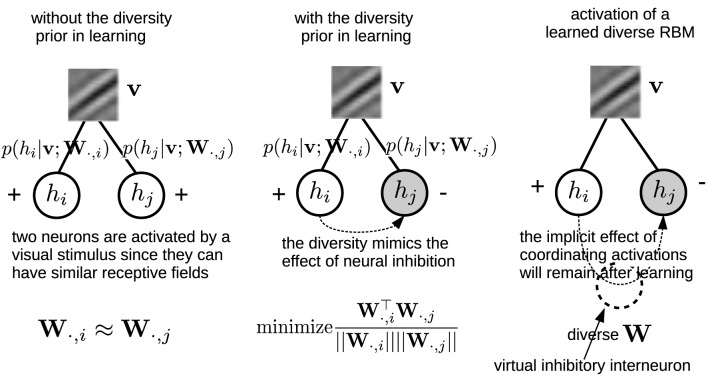
**Left, Middle:** An illustrating example shows how the diversity prior would mimic the effect of inhibition among neurons' activations during learning. Empty circles denote activated neurons while gray circles are inactivated ones. **Right**: although the diversity prior is only employed in the learning phase, its implicit effect on coordinating neurons' activations will remain after learning. Intuitively, it can be considered as if there would exist virtual inhibitory interneurons which are induced by the diversity prior.

Note that the denominator in Equation (11) is necessary, because eliminating it will generate many “dead” neurons. This repulsive design among **W**_·, *j*_ was also employed in the local competition algorithm (LCA) (Rozell et al., 2008). Zylberberg et al. (2011) also found that inhibition between two neurons are proportional to the similarity (measured by the vector dot product) between their receptive fields. Here, in order to gain a more clear understanding on how the diversity prior can replicate the effect of neural inhibition, an illustrating example is presented in Figure 3. In particular, for computing the gradient with respect to **W**, Equation (8) needs to infer the activations of the hidden units. The prior, which can bias the columns of **W** toward a more diverse population will indirectly coordinate the activations by suppressing the emergence of similar receptive fields, and therefore leads to a similar effect neural inhibition has during learning. Also, the effect from the prior will remain after learning with the learned diverse **W**. An extreme case is that the activation probabilities of neurons are exclusive to each other. Sparsity and selectivity are expected to be enhanced simultaneously by using this diversity-induced bias (Equation 11) (Figure 1B). We can define the prior probability distribution over parameters *p*(**W**) as

(12) $$p(W)\propto \exp\left(-\lambda \cdot \sum_{j=1}^{N_{h}}\sum_{k\neq j}^{N_{h}}{\left\|\frac{W_{\cdot ,j}^{⊤}W_{\cdot ,k}}{\left||W_{\cdot ,j}||||W_{\cdot ,k}|\right|}\right\|}^{2}\right).$$

Then, the parameters can be estimated via maximum a posteriori (MAP):

(13) $$\begin{array}{l} {\text{W}}^{*}=\arg\underset{\text{W}}{\max}p\left(\text{W}|𝒟\right)=\arg\underset{\text{W}}{\max}p\left(\text{W}\right)\overset{L}{\underset{l=1}{\prod }}\underset{\text{h}}{\sum }p\left({\text{v}}^{\left(l\right)},\text{h}|\text{W}\right) \end{array}$$

In our previous work (Xiong et al., 2014), we used absolute cosine similarities, of which the derivative cannot be analytically computed and therefore we had to resort to MCMC-based simulated annealing to conduct MAP. However, here by using the square cosine similarity, Equation (13) can be converted to a constrained concave optimization:

(14) $$\begin{array}{l} \begin{array}{ccc} {\text{W}}^{*} & = & \arg\underset{\text{W}}{\max}𝓛\left(𝒟\right)-\lambda \overset{N_{h}}{\underset{j=1}{\sum }}\overset{N_{h}}{\underset{k\neq j}{\sum }}{\left({\text{W}}_{\cdot ,j}^{⊤}{\text{W}}_{\cdot ,k}\right)}^{2}\\ s.t. & \forall j\in \left[1,N_{h}\right],||{\text{W}}_{\cdot ,j}||=1 \end{array} \end{array}$$

In this paper, since the above optimization problem is concave with respect to **W**, we employed gradient ascent to solve it (see the Appendix for details), and derived an iterative update of **W** as

(15) $$\begin{array}{l} W_{\cdot ,j}^{t+1}=W_{\cdot ,j}^{t}+\nabla_{W}\hat{𝓛}\left(𝒟\right)-2\lambda (\sum_{k\neq j}^{N_{h}}\left(W_{\cdot ,k}⊗W_{\cdot ,k}\right)\;\\ +\;C\frac{||W_{\cdot ,j}||-1}{\left||W_{\cdot ,j}|\right|}I_{N_{v}})W_{\cdot ,j} \end{array}$$

where ⊗ denotes the outer product between vectors, and **I**_*N*_*v*__ is a *N*_*v*_ × *N*_*v*_ identity matrix. In Equation (15) we can see that the iterative update of **W** is composed of two parts, where the first is the gradient of the log-likelihood while the second is the gradient of the log prior.

## 3. Results

In this section the learned receptive fields are shown, with which we measure the selectivity and sparsity of neurons' activations. We also compare the learned receptive fields with physiological data. Finally, we test the learned receptive fields in an image reconstruction experiment. The training dataset, the code of learning RBM, the learned diverse RBM and other materials used in our experiments are available at: https://iis.uibk.ac.at/public/xiong/resources.html#Diverse_RBM. Following Hinton (2002), we conducted training on mini-batches at one epoch. In all 400 epochs were run and it takes around 18 h with our Matlab code on an Intel core i7 laptop.

### 3.1. Basis images

**Figure 4:**
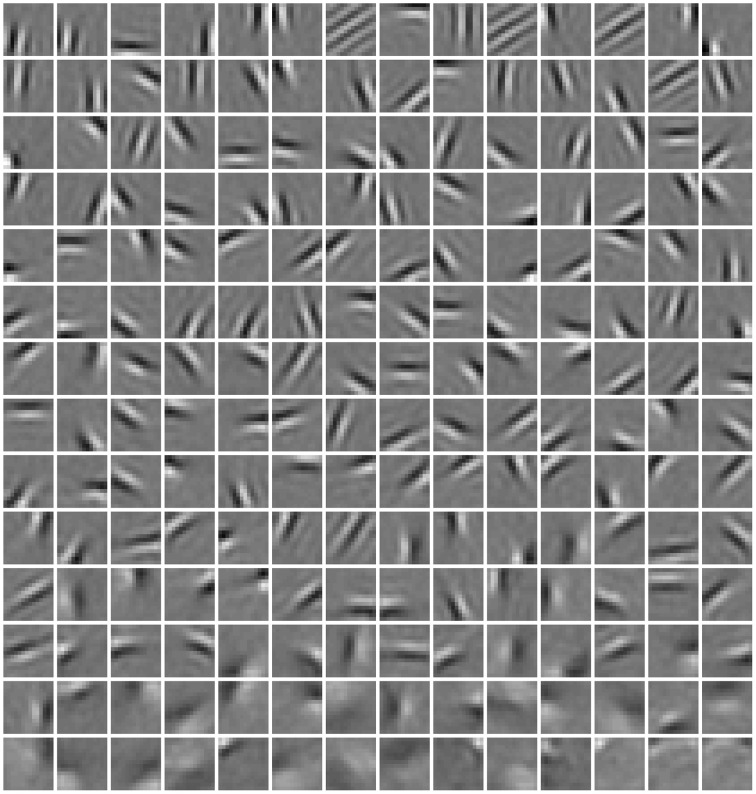
**Basis images (columns of W) learned in our model**. They can be also considered as receptive fields since we found that they are almost identical.

In Figure 4, a subset of basis images (i.e., columns of **W**) of RBMs trained with the diversity prior are shown. They look quite similar to the receptive fields of simple cells in macaque monkey V1 (Zylberberg et al., 2011, Figure 3). Rigorously speaking, basis images cannot be directly considered as receptive fields since they are internal connections or representations instead of response characteristics. The receptive fields of ICA are usually estimated as the inverse of the weight matrix (van Hateren and van der Schaaf, 1998), while in sparse coding reverse correlation is used for receptive fields (Olshausen and Field, 1996). Here, we employed a reverse correlation method similar to Hosoya (2012) who also developed a probabilistic model. For each hidden unit, its receptive field is estimated as

(16) $$\begin{array}{l} RF=\overset{S}{\underset{s=1}{\sum }}p\left(h_{j}=1|{\text{v}}_{s}\right){\text{v}}_{s}, \end{array}$$

where *p*(*h*_*j*_ = 1|**v**_*s*_) is computed as in Equation (2), while ${\left\{{\text{v}}_{s}\right\}}_{s=1}^{S}$ is a set of visual stimuli which are randomly selected in the training database. This is a little different from the procedure by Hosoya (2012), since they generate synthetic **v**_*s*_ from a Gaussian distribution. Meanwhile, we arrived at a finding similar to Hosoya (2012). By linearly fitting the *RF* of each unit to its corresponding basis images, we found that our basis images are almost identical to their corresponding receptive fields.

### 3.2. Selectivity and sparsity

There exist several ways to measure selectivity and sparsity, out of which *kurtosis* and *Treves-Rolls sparseness* are popularly used (Willmore and Tolhurst, 2001). Willmore and Tolhurst (2001) empirically proved that there exists a high correlation between these two measures. In other words, there would be no difference in using these two measures to quantify neurons' activations. Here, we use Treves-Rolls sparseness.

For a neuron, its selectivity is computed across all *L* input visual stimuli:

(17) $$selectivity\;=\;1-\frac{(\sum_{l=1}^{L}r_{l}/L{)}^{2}}{(\sum_{l=1}^{L}r_{l}^{2}/L)}$$

where *r*_*l*_ is the activation probability of the neuron given the *l*th stimulus, computed as in Equation (2).

**Figure 5:**
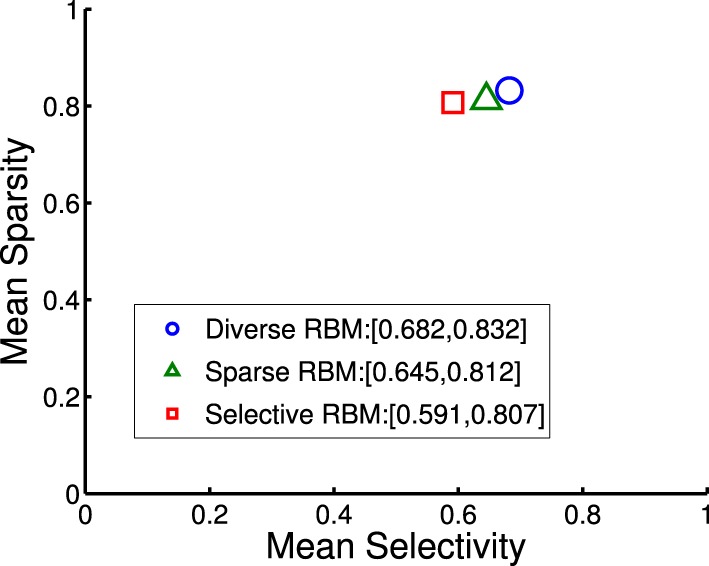
**Mean sparsity and mean selectivity of neurons' activations in diverse RBM, sparse RBM and selective RBM, respectively**.

**Figure 6:**
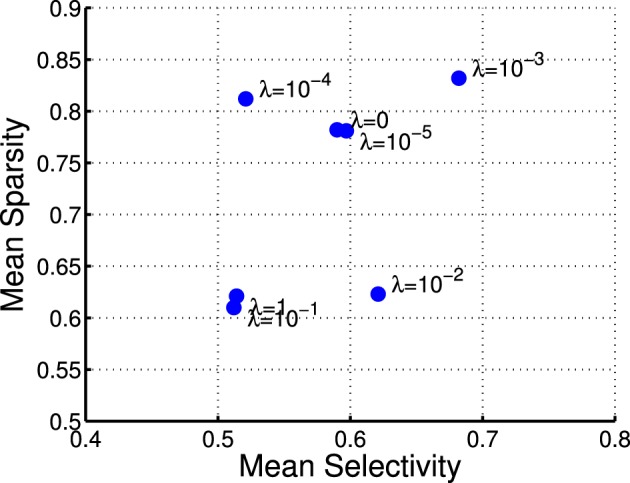
**Selectivies and sparsities when using different λ-values in the diverse RBM**.

**Figure 7:**
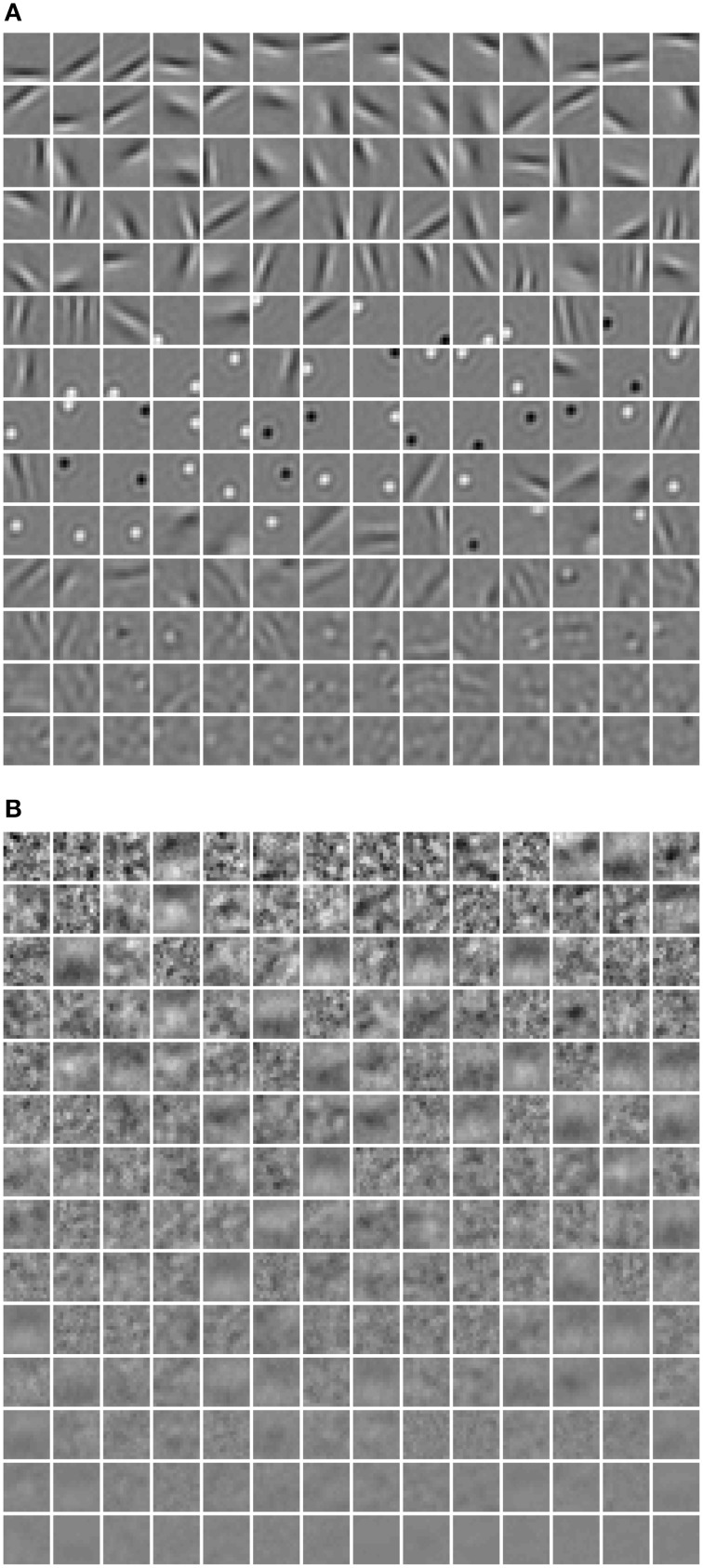
**The receptive fields learned using (A) λ = 10^−5^, (B) λ = 1**.

The sparsity of population activations by one stimulus is computed across all *N*_*h*_ neurons:

(18) $$sparsity\;=1-\frac{{\left(\sum_{j=1}^{N_{h}}r_{j}/N_{h}\right)}^{2}}{\left(\sum_{j=1}^{N_{h}}r_{j}^{2}/N_{h}\right)}$$

where *r*_*j*_ denotes the activation probability of the *j*th neuron by the stimulus. We computed the mean selectivity of all 200 neurons and the mean sparsity on all training small patches. The results are plotted in Figure 5. Two relevant models (selective RBM and sparse RBM, see Section 4.1) were tested as well for comparison. It can be seen that using the diversity prior in learning can result in comparable selectivity and sparsity as using selectivity prior or sparse prior. Meanwhile, the diversity prior should be preferred since it generates a much smaller number of “dead” neurons (see Section 4.1). In our experiment, λ = 10^−3^ was used to obtain the above result. To check how λ value affects sparsity and selectivity, in Figure 6 a plot with several λ is presented. When λ is small, e.g., 0, 10^−5^, 10^−4^, the effect of the diversity prior is weak or totally removed and both selectivity and sparsity decrease (Figure 6). The receptive fields of a diverse RBM trained with λ = 10^−5^ are shown in Figure 7A. If we use a big value of λ, e.g., 10^−2^, 10^−1^, 1, the iterative update of **W** Equation (15) is greatly dominated by the prior part, and therefore the fitness to the training data 𝒟 deteriorates. It can be seen that selectivity and sparsity also decrease (even to a larger degree) using relatively large λs (Figure 6). The receptive fields learned with λ = 1 are displayed in Figure 7B.

### 3.3. Comparison with biological data

**Figure 8:**
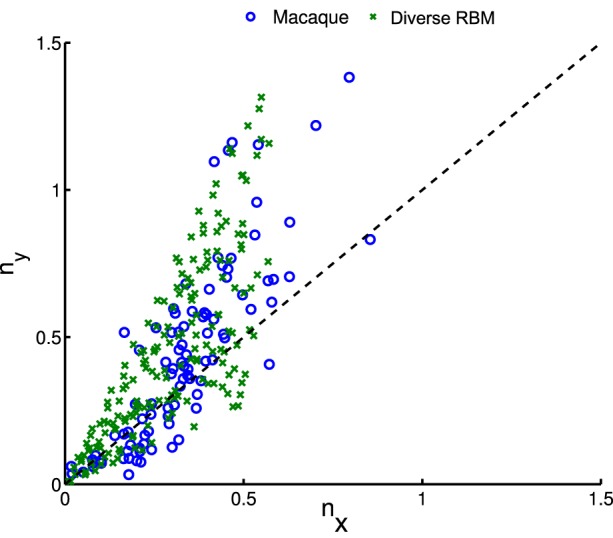
**After fitting receptive fields with Gabor filters, we pooled their shape profiles (***n***_***x***_, ***n***_***y***_), for comparison to physiological data of macaque monkeys (Ringach, 2002)**.

**Figure 9:**
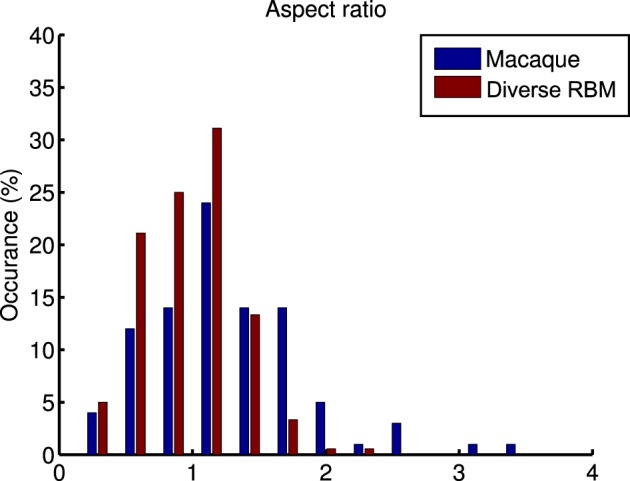
**A comparison of the histograms of aspect ratios (***n***_***x***_∕***n***_***y***_) within macaque monkey V1 neurons and the learned diverse RBM neurons**.

To better compare our receptive fields against physiological results (Ringach, 2002), we first fitted our receptive fields to Gabor filters:

(19) $$\begin{array}{l} \begin{array}{ccc} G\left(x,y;x_{0},y_{0},A,\sigma_{x},\sigma_{y},\theta ,f,\phi \right) & = & A\cos\left(2\pi fx^{\prime }+\phi \right)\\ \exp\left(-\frac{x^{\prime 2}}{2\sigma_{x}^{2}}-\frac{y^{\prime 2}}{2\sigma_{y}^{2}}\right)\\ x^{\prime } & = & \left(x-x_{0}\right)\cos\theta +\left(y-y_{0}\right)\sin\theta \\ y^{\prime } & = & -\left(x-x_{0}\right)\sin\theta +\left(y-y_{0}\right)\cos\theta \end{array} \end{array}$$

whose parameters are the center position (*x*_0_, *y*_0_), amplitude *A*, size (σ_*x*_, σ_*y*_), orientation θ, spatial frequency *f* and phase ϕ. The fitting is done via the *Nelder-Mead Simplex* method, and therefore is not very reliable. Similar to Hosoya (2012) and Zylberberg and DeWeese (2013), we conducted quality control by filtering out some receptive fields which were poorly fitted. First, we compared our receptive fields with those of macaque monkey V1 cells^2^ (Ringach, 2002) in units of the sinusoidal wavelength: (*n*_*x*_, *n*_*y*_) = (σ_*x*_*f*, σ_*y*_*f*). In Figure 8, we pooled (*n*_*x*_, *n*_*y*_) of our receptive fields as well as the data from Zylberberg and DeWeese (2013). We found that they don't deviate very much although they slightly differ from each other. We also checked the statistics of aspect ratios within receptive fields: $\frac{n_{y}}{n_{x}}$. In Figure 9 two histograms are displayed, which are global distributions of aspect ratios from our receptive fields and from the macaque monkey V1 cells, respectively. We can see that they are also quite close.

### 3.4. Image reconstruction

Reconstruction using RBMs is quite straightforward. First, small, non-overlapping patches (size 14 × 14) were extracted from a preprocessed image. For each small patch **v**, the activation probability of each neuron *p*(*h*_*j*_|**v**) can be computed as in Equation (2). Then, instead of using binary states of *h*_*j*_, *p*(*h*_*j*_|**v**) is used for recovering **v** by using Equation (3). It is worth noting that although RBMs are probabilistic models, we use the value of *p*(*v*_*i*_|**h**) to recover the intensity of each pixel and thus the reconstruction is deterministic.

**Figure 10:**
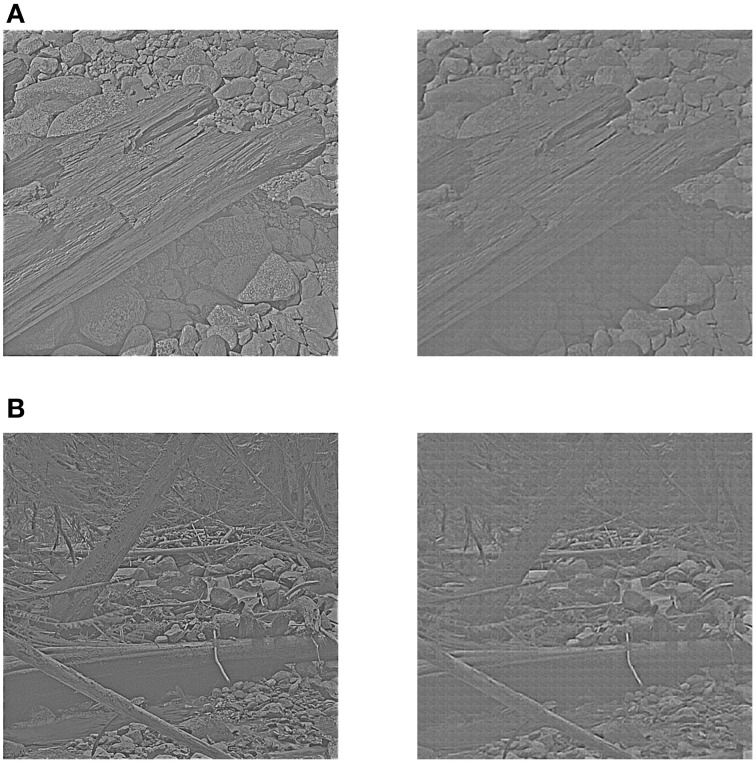
**Reconstruction using receptive fields of the learned diverse RBM**. The left panel of **(A,B)** shows two test images after preprocessing, while the right panel of **(A,B)** shows two corresponding reconstructions.

Out of the 10 images in the original database, 8 were used for training and the remaining 2 were used for testing the image reconstruction. The two test images were whitened and sigmoid-mapped using the same preprocessing procedure as the training images. They are shown in the left panel of Figure 10. In the right panel of Figure 10 the reconstructions of the two test images are presented. It can be seen that the reconstructions look very good in qualitative terms.

## 4. Discussion

### 4.1. Sparsity and selectivity prior on RBM

There are previous studies that learn simple cell receptive fields through the use of RBMs, either enforcing sparsity or selectivity. One recent example of the former is the sparse group restricted Boltzmann machine (SGRBM) (Luo et al., 2011), an RBM trained with the CD algorithm plus an *l*1∕*l*2 norm regularization on the activations of the neuron population. At each iteration, given a visual stimulus, and after computing the activation probabilities of the whole neuron set, SGRBM attempts to minimize the *l*1∕*l*2 norm of the set of activation probabilities. Although *l*1∕*l*2 norm regularization can ensure sparsity, it can also lead to many “dead” (never responding) and “potential over-tolerant” (always responding) neurons (see Figure 1D). In the case of the latter, a study that enforces selectivity is the one from Lee et al. (2007) which uses a selectivity-induced regularization that suppresses the average activation probability of each neuron to all training stimuli.

**Figure 11:**
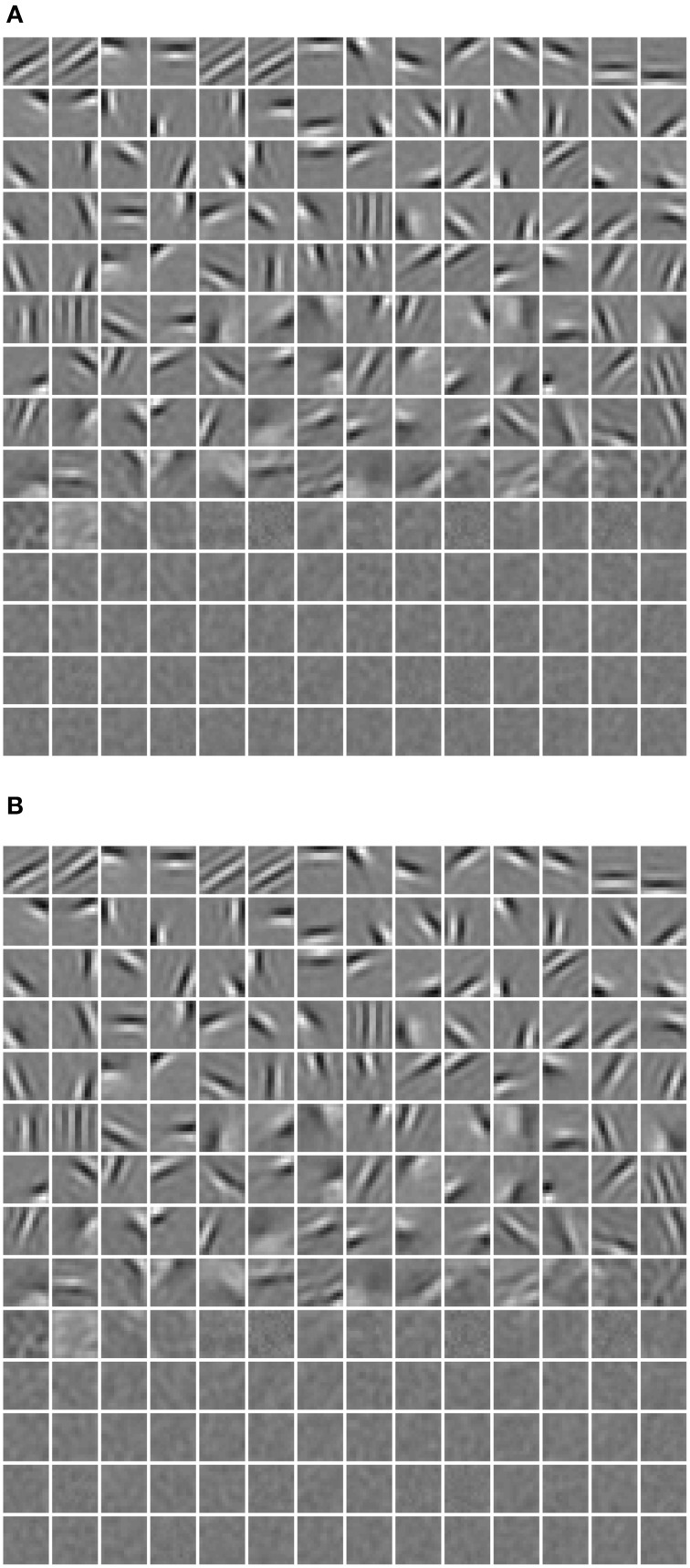
**The receptive fields learned in the learned (A) sparse RBM, (B) selective RBM**.

**Figure 12:**
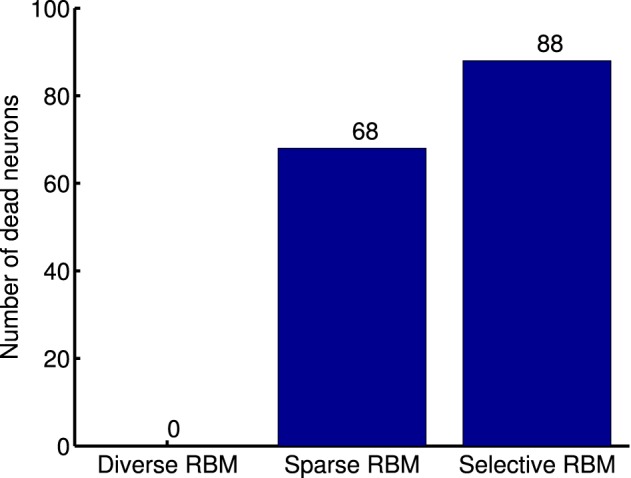
**Number of dead neurons in the learned diverse RBM, sparse RBM and selective RBM, respectively**.

One limitation of this strategy, as argued by Goh et al. (2010), is that decreasing average activation probabilities cannot guarantee selectivity. Instead, it will result in many similar neurons with uniformly low activation probabilities to all types of visual stimuli, which are prone to be “dead” as well. Following this line of thought and in order to prove the validity of our diverse RBM, two additional RBMs were trained using the CD algorithm with sparse regularization (sparse RBM) (Luo et al., 2011) and the CD algorithm with selectivity regularization (selective RBM) (Lee et al., 2007). For both of them, 200 hidden neurons were learned and their receptive fields are presented in Figure 11. We can see that the neurons' receptive fields learned in sparse RBM and selective RBM look similar to those of our RBM trained with a diversity prior. However, both sparse CD and selective CD led to many useless, “dead” neurons. We estimated the rough number of “dead” neurons by counting the number of neurons whose maximal activation probabilities to all training stimuli is smaller than 0.1, and the results are shown in Figure 12. Furthermore, we also computed the mean selectivity and the mean sparsity of neurons in sparse RBM and selective RBM in the same way as we did for the diverse RBM; their results are also shown in Figure 5.

### 4.2. The equivalent to a diversity prior in biological systems

Knowing about how neuron receptive field properties arise is of great importance in visual neuroscience in order to hypothesize the circuits and connections that give rise to those properties. On one hand, one of the characteristics of simple cells in V1 is selectivity to oriented stimuli. These can be obtained through placing some constraint in learning from natural images. An example is the influential work by Olshausen and Field (1996). A set of coefficients is then formed such that they have a cost associated to them depending on how the activity is distributed. The aim is to increase sparsity, meaning lower cost. This approach leads to V1-like simple-cell receptive fields through the learning of a set of weights that correspond to the connections of the input layer with simple neurons in area V1.

On the other hand, inhibition seems to play a central role in the shaping of simple-cell receptive fields. We can consider three types of inhibitory inputs: feedforward, lateral and feedback (also known as recurrent). Feedforward inhibition is regarded as the main source of orientation selectivity in simple cells by some researchers (Heggelund, 1981; Celebrini et al., 1993; Ferster and Miller, 2000) and has been modeled by others, e.g., (Azzopardi et al., 2014). The classical role of feedback connections was the enhancement of receptive-field responses to top-down modulations (Ito and Gilbert, 1999; Treue, 2003), which have been successfully modeled for attention (Rodriguez-Sanchez et al., 2007) and contour integration (Neumann and Sepp, 1999; Tschechne and Neumann, 2014). But other studies are in support of feedback connections as the source of simple-cell selectivity through recurrent connections, most recently from Angelucci and Bressloff (2006). The appearance of orientation selectivity this way has also been proposed in models of recurrent inhibition, e.g., (Sabatini, 1996; Carandini and Ringach, 1997). Finally, even though there is an alive discussion regarding if orientation selection is achieved through feedforward or recurrent connections, it is interesting to note that none of them rule out that lateral inhibition can at least be partially blamed for this selectivity, e.g., (Celebrini et al., 1993; Angelucci and Bressloff, 2006). Lateral connections have in fact being made explicit into recent sparse coding models (Garrigues and Olshausen, 2008; King et al., 2013).

The common ground of all the aforementioned works is that inhibition is fundamental to the selectivity properties of simple cells, irrespective of where that inhibition comes from. Inhibition is also linked to the appearance of sparse sensory coding (Vinje and Gallant, 2000; Haider et al., 2010). We can conclude then, that inhibition would generate RF diversity, since as we have shown in this work (Figure 1), imposing diversity generates both selective and sparse neural populations. By explicitly favoring diversity in our model, we would be mimicking the effect that inhibition should have on feature learning in a biological system.

## 5. Conclusion

We test a recent new concept, that of diversity (Padmanabhan and Urban, 2010; O'Donnell and Nolan, 2011), by applying diversification on the columns of **W** when using a RBM to learn receptive fields. This diversification has the implication of providing a set of neurons that is at the same time sparse and selective, which, as mentioned in the introduction, is not always the case for sparse models. Imposing diversity is thus a more general condition to achieve both, sparsity and selectivity.

### Conflict of interest statement

The authors declare that the research was conducted in the absence of any commercial or financial relationships that could be construed as a potential conflict of interest.

## Footnotes

^1^Available on http://redwood.berkeley.edu/bruno/sparsenet/.

^2^Data are available at: http://www.ringachlab.net/lab/Data.html.

## Appendix

### A1. MAP optimization with the sum similarity minimization prior

The optimization problem (Equation 14) can be rewritten as

(A1) $$\begin{array}{l} \underset{\text{W}}{\max}𝓛\left(𝒟\right)-\lambda \underset{O}{\underset{︸}{\left[\overset{N_{h}}{\underset{j=1}{\sum }}\overset{N_{h}}{\underset{k\neq j}{\sum }}{\left({\text{W}}_{\cdot ,j}^{⊤}{\text{W}}_{\cdot ,k}\right)}^{2}+C\overset{N_{h}}{\underset{j=1}{\sum }}{\left(||{\text{W}}_{\cdot ,j}||-1\right)}^{2}\right]}}, \end{array}$$

where *C* is an extra parameter that is set relatively large to guarantee the satisfaction of the constraints in Equation (14). In our experiment, *C* is equivalent to 10^4^. In this way, the constrained optimization problem is converted to an unconstrained one. It was already shown that the gradient ascent can used to maximize 𝓛(𝒟). It is easy to see that *O* is also convex with respect to **W**; therefore, the same gradient ascent can be also applied on −λ*O*. The gradient of *O* with respect to **W** can be computed as

(A2) $$\begin{array}{rcl} \frac{\partial O}{\partial {\text{W}}_{\cdot ,j}} & = & 2\overset{N_{h}}{\underset{k\neq j}{\sum }}\left({\text{W}}_{\cdot ,j}^{⊤}{\text{W}}_{\cdot ,k}\right){\text{W}}_{\cdot ,k}+2C\left(||{\text{W}}_{\cdot ,j}||-1\right)\frac{{\text{W}}_{\cdot ,j}}{\left||{\text{W}}_{\cdot ,j}|\right|} \end{array}$$

(A3) $$\begin{array}{rc} = & 2\overset{N_{h}}{\underset{k\neq j}{\sum }}\left({\text{W}}_{\cdot ,k}⊗{\text{W}}_{\cdot ,k}\right){\text{W}}_{\cdot ,j}+2C\left(||{\text{W}}_{\cdot ,j}||-1\right)\frac{{\text{W}}_{\cdot ,j}}{\left||{\text{W}}_{\cdot ,j}|\right|} \end{array}$$

(A4) $$=2\left(\sum_{k\neq j}^{N_{h}}(W_{\cdot ,k}⊗W_{\cdot ,k})+C\frac{||W_{\cdot ,j}||-1}{\left||W_{\cdot ,j}|\right|}I_{N_{v}}\right)W_{\cdot ,j},$$

where ⊗ denotes the outer product between vectors and **I**_*N*_*v*__ is a *N*_*v*_ × *N*_*v*_ identity matrix.
